# The College Programme

**Published:** 1919-01-04

**Authors:** 


### The College Programme.

SIR,—I have read with interest the article in this week's HOSPITAL by "Ierne." It voices what I and many others in the profession feel. It is quite time that nurses had shorter hours and were paid a living wage—one in keeping with the standard of education which is desired in the nurses of to-day. As to saving for our old age, on the salary paid in civil institutions, it is quite impossible. In many of our hospitals the night duty consists of twelve hours, and as everyone knows, nursing sick people entails both mental and physical strain.

Is there any wonder that nurses are too weary and tired when they come off duty to take proper recreation and so become self-centred and dissatisfied?

I trust that the College will see that we get (what every worker of to-day expects) time and energy to make life outside interesting. That will bring us back to our work fresher and more fit mentally and physically to help those who are so dependent on our ministrations, and the spirit in which they are carried out.

A MEMBER OF THE COLLEGE OF NURSING.

